# A Field Trial of Alternative Targeted Screening Strategies for Chagas Disease in Arequipa, Peru

**DOI:** 10.1371/journal.pntd.0001468

**Published:** 2012-01-10

**Authors:** Gabrielle C. Hunter, Katty Borrini-Mayorí, Jenny Ancca Juárez, Ricardo Castillo Neyra, Manuela R. Verastegui, Fernando S. Malaga Chavez, Juan Geny Cornejo del Carpio, Eleazar Córdova Benzaquen, César Náquira, Robert H. Gilman, Caryn Bern, Michael Z. Levy

**Affiliations:** 1 Department of International Health, Johns Hopkins Bloomberg School of Public Health, Baltimore, Maryland, United States of America; 2 Facultad de Ciencias y Filosofía, Universidad Peruana Cayetano Heredia, Lima, Peru; 3 Department of Epidemiology, Johns Hopkins Bloomberg School of Public Health, Baltimore, Maryland, United States of America; 4 Dirección Regional del Ministerio de Salud, Arequipa, Peru; 5 Departamento de Microbiología y Patología, Facultad de Medicina, Universidad Nacional de San Agustín, Arequipa, Peru; 6 Division of Parasitic Diseases, Centers for Disease Control and Prevention, Atlanta, Georgia, United States of America; 7 Center for Clinical Epidemiology and Biostatistics, Department of Biostatistics and Epidemiology, University of Pennsylvania School of Medicine, Philadelphia, Pennsylvania, United States of America; Universidad de Buenos Aires, Argentina

## Abstract

**Background:**

Chagas disease is endemic in the rural areas of southern Peru and a growing urban problem in the regional capital of Arequipa, population ∼860,000. It is unclear how to implement cost-effective screening programs across a large urban and periurban environment.

**Methods:**

We compared four alternative screening strategies in 18 periurban communities, testing individuals in houses with 1) infected vectors; 2) high vector densities; 3) low vector densities; and 4) no vectors. Vector data were obtained from routine Ministry of Health insecticide application campaigns. We performed ring case detection (radius of 15 m) around seropositive individuals, and collected data on costs of implementation for each strategy.

**Results:**

Infection was detected in 21 of 923 (2.28%) participants. Cases had lived more time on average in rural places than non-cases (7.20 years versus 3.31 years, respectively). Significant risk factors on univariate logistic regression for infection were age (OR 1.02; p = 0.041), time lived in a rural location (OR 1.04; p = 0.022), and time lived in an infested area (OR 1.04; p = 0.008). No multivariate model with these variables fit the data better than a simple model including only the time lived in an area with triatomine bugs. There was no significant difference in prevalence across the screening strategies; however a self-assessment of disease risk may have biased participation, inflating prevalence among residents of houses where no infestation was detected. Testing houses with infected-vectors was least expensive. Ring case detection yielded four secondary cases in only one community, possibly due to vector-borne transmission in this community, apparently absent in the others.

**Conclusions:**

Targeted screening for urban Chagas disease is promising in areas with ongoing vector-borne transmission; however, these pockets of epidemic transmission remain difficult to detect *a priori*. The flexibility to adapt to the epidemiology that emerges during screening is key to an efficient case detection intervention. In heterogeneous urban environments, self-assessments of risk and simple residence history questionnaires may be useful to identify those at highest risk for Chagas disease to guide diagnostic efforts.

## Author Summary

In the wake of emerging *T. cruzi* infection in children of periurban Arequipa, Peru, we conducted a prospective field trial to evaluate alternative targeted screening strategies for Chagas disease across the city. Using insect vector data that is routinely collected during Ministry of Health insecticide application campaigns in 3 periurban districts of Arequipa, we separated into 4 categories those households with 1) infected vectors; 2) high vector densities; 3) low vector densities; and 4) no vectors. Residents of all infected-vector households and a random sample of those in the other 3 categories were invited for serological screening for *T. cruzi* infection. Subsequently, all residents of households within a 15-meter radius of detected seropositive individuals were invited to be screened in a ring case-detection scheme. Of 923 participants, 21 (2.28%) were seropositive. There were no significant differences in prevalence across the 4 screening strategies, indicating that household entomologic factors alone could not predict the risk of infection. Indeed, the most predictive variable of infection was the number of years a person lived in a location with triatomine insects. Therefore, a simple residence history questionnaire may be a useful screening tool in large, diverse urban environments with emerging Chagas disease.

## Introduction

Chagas disease has historically occurred in poor rural settings of Latin America [1], [2], [3]. In the rural areas of the Department of Arequipa in southern Peru, reports of Chagas disease and its vectors date back to the early 20^th^ century [4], [5], [6], where the disease has persisted in an endemic state [7]. However, recent case reports [8] and epidemiologic studies [9], [10] have documented emerging vectorial transmission of *Trypanosoma cruzi*, the etiologic agent of Chagas disease, in communities of the urban capital of Arequipa. As *T. cruzi* and *Triatoma infestans*, the sole insect vector in this setting, spread through the city of Arequipa (pop. 864,250) [11], [12], several hundred thousand people are at risk of infection.

Most individuals have mild or no symptoms during the acute phase of Chagas disease, and pass into the chronic phase without having the infection detected. In the chronic phase, most infected individuals show no signs or symptoms and are considered to have the indeterminate form of the disease. An estimated 20–30% of infected individuals will later develop the cardiac or digestive forms of chronic Chagas disease. Advanced cardiac or digestive disease cannot be reversed and can be fatal [13], [14], [15]. However, for patients with the indeterminate form or early cardiomyopathy, antitrypanosomal treatment is reported to decrease the probability of disease progression [15].

It is important to detect individuals with chronic *T. cruzi* infection because they need clinical attention, may be candidates for treatment, and pose risk for further transmission – either vectorial, congenitally or through blood donation [16], [17], [18]. Diagnostics for Chagas disease in southern Peru are expensive relative to local income and the limited government budget for vector-borne disease control [19]. Alarmed by the urban encroachment of *T. infestans*, the Arequipa regional Ministry of Health (MOH) recommends universal testing of all residents of communities with *T. cruzi*-infected *T. infestans*. However, such wide-scale testing is too costly for practical implementation. Population screening is further challenged by the very low sensitivity of field-applicable rapid tests in Arequipa, and screening must employ high sensitivity conventional tests such as enzyme-linked immunosorbent assays [20]. Given the large population of Arequipa city and limited health resources, targeted interventions are the only viable option to screen for chronic *T. cruzi* infection in this population.

Although mass vector-control campaigns have had success in Latin America [21], [22], numerous authors call for improved screening strategies and expansion of treatment for persons with Chagas disease [19], [23], [24], stressing the importance of cost-efficiency, sustainability, and integration of sectors [22], [25]. Several cost-effectiveness studies of Chagas disease interventions have focused on devising optimal insecticide application and blood donor testing schemes [25], [26], [27], [28], but few have examined strategies for human serologic testing in endemic or epidemic areas. A study by Mott *et al.* [29] indicated the potential for targeted screening around detected positive children under 5 years of age in a rural area of Brazil. Gurtler *et al.* [19], [30] highlight the potential efficiency of linking serologic screening to vector control campaigns. It remains unclear how to implement cost-effective, targeted screening programs across a large and diverse urban environment with dynamic vector infestation.

In one periurban community of Arequipa that was heavily infested with *T. infestans*, 5.3% of children had *T. cruzi* infection [31]. Age-prevalence curves of infection [10] and the spatial distribution of cases suggested that the disease was in an epidemic phase in this community [31]. A retrospective analysis of household vector data carefully collected during an insecticide-application campaign prior to serologic testing, indicated that a two-step targeted screening intervention based on household entomologic risk factors and ring testing around identified cases would have captured 83% of infected children, while minimizing the testing of negative children [31]. In the present operational research study, we test 4 targeted screening strategies based on similar household entomologic data, and one adaptive spatial strategy, in 18 periurban communities of Arequipa, Peru. We compare the performance and cost of each, and evaluate the patterns of infection they reveal. Our objective was to test the operational feasibility of routinely collected data from vector control campaigns for human Chagas disease detection on a larger urban scale.

## Methods

### Study setting

This cross-sectional study was conducted in 3 districts of the city of Arequipa. Each district is composed of several communities, which span a gradient of development. In general, there are higher quality housing materials and infrastructure in the parts closer to the urban center and more recent, less developed settlements towards the periphery.

As part of a coordinated Chagas control campaign, Ministry of Health (MOH) vector control teams applied two rounds of deltamethrin insecticide (5% Wettable Powder; K-Othrine, Bayer; target dose of 25 mg a.i./m^2^), spaced six months apart, to houses in 92 communities across the 3 study districts between 2005 and 2009. The insecticide has an immediate repellent effect, and a delayed lethal effect, on the triatomine bugs. Technicians were trained by the MOH to collect as many emerging triatomine bugs as possible from each house at the time of spraying, thereby obtaining a sample of vectors infesting the house. All technicians were overseen by a brigade chief experienced in Chagas disease vector control. The insects were placed in containers coded by household and delivered to the study laboratory in Arequipa for microscopic examination for *T.cruzi*, as described previously [9]. Of the 92 communities participating in the insecticide campaign, *T. cruzi*-infected triatomines were detected in only 18 communities (19.6%, 95% CI: 11.5–27.6). We limited our targeted screening strategies to residents of these 18 communities in which the etiologic agent of Chagas disease had been documented.

### Selection of households

We tested the following four targeted and mutually exclusive strategies for use in the initial screening step, based on household vector data collected at the time of the MOH spray campaign in the 18 communities. Each strategy grouped households on a gradient of risk for human *T. cruzi* infection based on information from prior studies [10], in the order listed below.

- *Infected vector*: Houses with *T. cruzi*-infected *T. infestans* (100% of eligible houses approached)
- *High vector density, uninfected*: Houses with only uninfected vectors collected in quantities at or above the 90^th^ percentile of the total vector count specific to that house's community (25% of eligible houses approached, randomly selected)
- *Low vector density, uninfected*: Houses with only uninfected vectors collected in quantities below the 90^th^ percentile of the total vector count specific to that house's community (1% of eligible houses approached, randomly selected)
- *Uninfested*: Houses in which no *T.infestans* were detected at the time of spraying (1% eligible houses approached, randomly selected)

**Figure 1:**
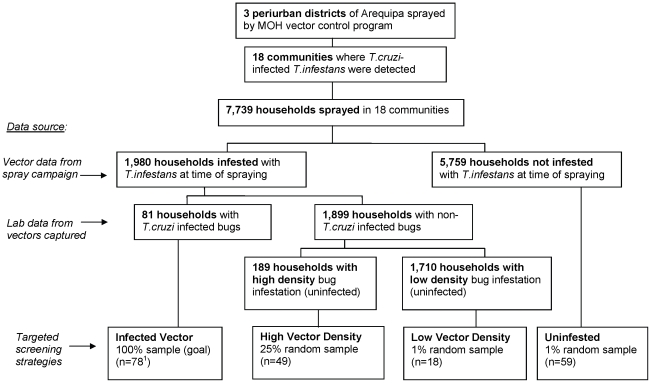
Flowchart of household selection into targeted screening strategies for Chagas disease based on vector-control data. Figure 1 displays the scheme used to stratify households into 4 targeted strategies to screen household members for human *T. cruzi* infection. The stratification was based on Ministry of Health (MOH) entomologic data from recent spray campaigns and laboratory analysis in the city of Arequipa, Peru. ^1^ Although 81 households had *T.cruzi*-infected *T.infestans* at the time of spray, 1 household was no longer inhabited at the time of this study, and 2 refused to participate.

Figure 1 presents a flowchart of the process by which vector data were used to separate the sprayed households into each targeted screening strategy. Sampling percentages for each strategy were chosen to stay within the sample size of the study. Random selection of houses was carried out using a random number generator code in Stata 10 (StataCorp) applied to a list of all houses in each category.

Following this initial screening step, the second step consisted of adaptive ring sampling in which testing was offered to the inhabitants of all houses within 15 meters of a seropositive individual detected through one of the above strategies. City block layouts are such that houses are contiguous (share one or more walls). A radius of 15 meters would allow to capture, on average, 5 immediate neighbors per index house (two lateral neighbors, one neighbor behind the house and two diagonal neighbors). We subsequently tested another 15 meter radius around any secondary seropositive individuals detected during the adaptive sampling.

There had been no insecticide-application campaigns in any of the study sites prior to the 2005–2009 campaigns described here. Houses that were not sprayed during the vector-control campaign could not be assigned to the vector-based strategies, and were excluded. However, they were eligible for the adaptive ring testing. Within the randomized strategies, households that refused to participate in the serology were replaced by additional random selections until reaching the enrollment goal for each strategy. Refusal events were recorded and some reasons for refusal noted. Household participation was defined as participation by at least 1 member. Households were mapped using GoogleEarth (Google Inc.) and ArcGIS 9.3.1 (ESRI, 1999–2008).

### Ethics statement

The study was approved by the human subjects research ethics committees of Johns Hopkins Bloomberg School of Public Health and by the Universidad Peruana Cayetano Heredia in Peru. Signed informed consent was obtained prior to participation by all adults and the parents of all participating children <18 years. In addition to their parent's consent, children ≥7 years old provided signed informed assent prior to participating.

### Recruitment of participants

Trained field workers approached each selected house and explained the entire study. In addition, they informed residents about the specific vector data obtained from their house during the spray campaign. For example, residents of households in the infected vector strategy were informed that triatomines collected from their homes carried the parasite that causes Chagas disease. Each house's vector data was kept confidential and not shared with any other household; potential participants were only informed of the community-level risk, for example that *T. cruzi* carrying bugs had been found in other homes of their community. All individuals ≥1 year old were invited to participate.

### Data collection & management

Field workers conducted a brief household census with one adult member per house, and a questionnaire about demographics and exposure to Chagas disease risks with each individual study participant. Demographic variables included sex, age, and education level. Exposure variables focused on the residential history of participants. Starting with their place of birth, participants were asked to list and categorize each previous place of residence (stays longer than 1 month) as rural, urban or periurban, and to recall the presence or absence of “*chirimachas*”, the local name for *T. infestans*. Participant recall was aided by the fact that *T. infestans* is the sole Chagas disease vector in southern Peru and by the heightened awareness resulting from widespread radio, print and interpersonal messaging during the spray campaigns. This element of the questionnaire was not designed to test participant knowledge or ability to identify *T. infestans*, but to examine if recall through a simple questionnaire could be operationally useful to identify infected or high-risk individuals. In addition, for the purposes of informing future feasibility of this type of screening, we collected data on the costs of fieldwork for obtaining blood samples, specimen processing and testing, data management and quality control. All data was double digitated and managed in Microsoft Access®. We logged person-hours for each of these activities, the number of visits made to each house, and the cost of materials and overhead. This data would yield a simple estimate of the cost of screening strategy implementation for operational purposes.

### Specimen collection, processing and diagnostic testing

A venous blood specimen (3 ml for children younger than 5 years, 5 ml for participants 5 years or older) was collected from each consenting participant and transported to a Chagas disease field laboratory of the Universidad Peruana Cayetano Heredia (UPCH) located in Arequipa. At the field laboratory, specimens were centrifuged, aliquoted, and stored at −20°C until the time of diagnostic testing using the Chagatek ELISA kit following the manufacturer's instructions for cutoffs (BioMérieux). 100% of specimens with positive results by ELISA and a randomly selected 10% with negative results were tested using immunofluorescence assay (IFA) according to published methods [20]. Aliquots of the same specimens were processed in parallel in the UPCH Microbiology Laboratory in Lima for quality control. Individuals with positive results by both ELISA and IFA were considered to have confirmed infection. Those with positive ELISA results and negative IFA results were considered discordant. However, previous research has shown that ELISA-positive, IFA-negative specimens likely indicate true infection in Arequipa [32], and therefore for the purposes of our data analysis only we considered individuals with discordant test results as *T. cruzi* infected. Regardless of this grouping, all ELISA-positive participants were referred to the MOH along with their lab results, for a case-by-case evaluation of infection status and clinical management by physicians based on national guidelines.

### Statistical analysis

Participants' demographic and exposure variables described above were used to calculate frequencies and means, and to fit regression models on the outcome of detected *T. cruzi* infection. The total number of lifetime locations each participant had lived in was tabulated from the migration histories, as were each participants' total number of years lived in a rural, periurban, or urban locations, and in a location recalled as being infested with *T. infestans* (regardless of rural, urban or periurban). Because rates of infection were low in our sample, we used Poisson regression to evaluate associations between covariates and infection status using the prevalence ratios (PrR) [33], [34]. Since the data were neither over- nor under-dispersed with respect to the outcome, no adjustments were required [34]. We fit models with a random effect term (gamma distributed) to consider correlation among participants of the same household. For the regression analyses, education level was considered only for adults. All variables with p<0.2 in univariate regression analysis were considered in fitting a multivariate model. Statistical tests were conducted using Stata 10 (StataCorp).

## Results

At the time of insecticide application in our 18 study communities, 1980 of 7739 (25.6%, 95% CI: 24.6–26.6) sprayed households were found to be infested with triatomine vectors, with technicians capturing between 1–301 *T. infestans* per household. The 90^th^ percentile cut-off between the high-density and low-density vector houses was determined separately for each community and ranged from 11–85 *T. infestans* captured per household. Out of the 1980 households in which vectors were detected, eighty-one (4.1%) had vectors carrying *T. cruzi*. In total, 923 people from 249 households participated in the serological testing across the 18 communities in 3 districts. There were no statistically significant differences in demographics by district (data not shown). Participants had a mean age of 34 years (range: 1–94), and 78.9% were adults of age 18 or over. Of the total participants, 21 (2.28%) tested positive according to ELISA, and were considered *T. cruzi* infected for this analysis. Nineteen of these also had positive results by IFA, while 2 did not. All 21 infected individuals were over the age of 18.

Table 1 displays the results across strategies. Neither demographic characteristics nor infection prevalence differed significantly among participants recruited through the four alternative strategies. Although not statistically significant, the highest prevalence was observed among those screened in houses with no infestation detected (3.07%). However, we encountered high refusal rates during participant recruitment from the houses with no infestation detected. Of 97 households approached, only 59 (61%) participated. By contrast, 98% of households with *T. cruzi*-infected vectors detected participated in the study. In addition, the within household participation rate was lower for households in which no infestation was detected versus households in the infected vector group, 55% versus 70% participation, respectively. These data suggest a self-selection bias in participation among people living in houses with no infestation detected. Potential reasons for this are explored in the discussion.

**Table 1:** Comparison of targeted strategies to detect *T. cruzi*-infected individuals in 18 periurban communities, Arequipa, Peru.

Strategy name	Infected vector	High vector density	Low vector density	Uninfested (no infestation detected)	Adaptive ring testing
**Description**	Houses in which *T. cruzi* was observed in at least 1 vector collected during the spray campaign	Houses in the 90^th^ and above percentile of vector density in each community	Houses below the 90^th^ percentile of vector density in each community	Houses in which no vectors were detected at the time of spray	Houses within 15 m of a confirmed human infection detected through previous strategies
**Total number of households**	80^1^	189	1710	5759	68^2^
**Enrollment goal**	80 (100%)	49 (25% random sample)	18 (1% random sample)	59 (1% random sample)	68 (100%)
**Participating households of total approached** 3	78/80 (98%)	49/67 (73.1%)	18/21 (85.7%)	59/97 (61%)	45/68 (66.2%)
**Mean number of participants per household**	4.0	4.7	4.3	2.7	3.5
**Household member participation rate out of total members** 4	70.3%	63.6%	72.3%	55.0%	62.0%
**Prevalence of ** ***T. cruzi*** ** infection among study participants**	8/308 (2.60%)	2/209 (0.96%)	2/85 (2.35%)	5/163 (3.07%)	4/158 (2.53%)
**Average number of household visits per participant enrolled**	1.32	1.41	1.48	2.63	1.78
**Person-hours of fieldwork, per participant enrolled**	1.98	2.12	2.22	3.95	2.67

1 Originally 81 houses had *T.cruzi*-infected triatomines, however 1 house was no longer inhabited at the time of this study.

2 27 additional households within 15 meters of an index human infection had already been approached under the previous strategies.

3 Number of participating households out of total households invited to participate.

4 Percent of household members participating of total household members >1 year old.

The 923 participants had lived in a mean of 2.5 locations (range:1–15). Mean residence in a periurban location was 26.97 years (CI 25.98, 27.97), while mean residence in an urban location was 3.61 years (CI 3.11, 4.12), and 3.39 years (CI 2.91, 3.88) in a rural location. The *T. cruzi* infected human cases we detected had lived longer, on average, in rural places than non-cases (mean 7.20 years vs. 3.31, respectively; p = 0.0184).

The results of univariate analysis on *T. cruzi* infection status are shown in Table 2. The probability of infection with *T. cruzi* increased by 2% per year of age (p = 0.02), by 2% per year lived in a periurban location (p = 0.185), by 4% per year lived in a rural location (p = 0.04), and by 4% per year lived in a place with triatomine bugs (p = 0.008). Multivariate models with combinations of these variables did not fit the data better than the univariate models (data not shown). Given inherent dependence of all four of the above variables on calendar time, it is likely that all are describing a similar experience of risk.

**Table 2:** Univariate Poisson regressions^1^ on *T. cruzi* infection among targeted screening participants in Arequipa, Peru.

Variable	Percent or Mean (95% CI)	Prevalence Ratio (PrR)	P-value	N
**Male**	42.0% (38.8, 45.2)	1.03	0.939	923
**Completed primary school** 2	91.1% (89.0, 93.1)	0.94	0.924	728
**Age**	34.0 yrs (32.8, 35.1)	1.02	0.041	923
**Lifetime number of places lived** 3	2.5 places (2.3, 2.6)	1.06	0.585	923
**Years lived in rural locations** 3	3.4 yrs (2.91, 3.88)	1.04	0.022	923
**Years lived in periurban locations** 3	27.0 yrs (25.98, 27.97)	1.02	0.185	923
**Years lived in urban locations** 3	3.6 yrs (3.11, 4.12)	1.00	0.951	923
**Years lived in locations infested with ** ***Triatoma infestans*** 3	21.9 yrs (20.9, 22.9)	1.04	0.008	923

1 Poisson regressions included a random effect term to control for correlation among participants from the same household.

2 Univariate analysis of education includes only adults (age 18 or over).

3 The lifetime total number of places in which each participant had lived was tabulated from the migration histories, as were each participants' total number of years lived in a rural, periurban or urban location, and in a location recalled as being infested with *T. infestans*.

Table 1 also displays the results of the secondary case detection by 15 meter radii. The adaptive ring sampling of houses within 15 meters of the houses of the 17 index cases yielded 4 additional cases out of 158 individuals tested. Although employed in seven different communities, all four of the secondary detected cases lived in the same community, Simón Bolívar. Of the 10 total cases found in Simón Bolívar, 7 had lived only in urban districts of Arequipa, and 5 only in Simón Bolívar itself, suggesting a local, urban site of transmission.

The estimated costs for each study activity are shown in Table 3. The fixed overhead cost per participant was $7.14, including field materials, telecommunications, transportation, data management, and diagnostic testing, and this was standard across all strategies. However, the recruitment cost per participant varied greatly across strategies, being a function of both the sampling framework and differential participation rates for each. For the households with infected-vectors detected, our field team made 1.32 household visits per participant recruited, versus 2.63 visits to houses in which no infestation was detected, reflecting the higher refusal rate of the latter. Each household visit required an average of 1.54 person-hours, at which rate, the testing in houses where no infestation was detected was the most costly strategy per participant ($14.62 USD), and the infected-vector houses strategy the least costly per participant ($10.91 USD).

**Table 3:** Total expenditures to conduct targeted screening for *T. cruzi* infection in Arequipa, Peru, all strategies.

Activity^1^		Cost of activity per study participant^2^
**Fieldwork**	Person-hours spent on household visits for recruitment and study enrollment *(variable by strategy)*	Min: $3.77 (Infected vector); Max: $7.48 (No infestation detected)
	Materials, transportation, and telecommunications	$1.47
**Data management**	Person-hours for double data entry	$1.28
**Laboratory work**	Diagnostic test materials and reagents	$3.00
	Person-hours for human specimen processing, diagnostic and confirmation testing	$1.39
**Total cost per study participant**		Min: $10.91 (Infected vector); Max: $14.62 (No infestation detected)

1 Total expenditure based on the salary for fieldworkers, data managers, or lab workers, respectively, at the time of the study.

2 Exchange rate of 3.00 Peruvian Nuevo Soles (PEN) to the U.S. dollar (USD).

## Discussion

Chagas disease is a growing problem in Arequipa, Peru. A previous study in the city uncovered micro-epidemics of transmission associated with high density of vectors, suggesting that targeting screening based on entomologic information could be an efficient means of detecting *T. cruzi* infected individuals [31]. In this prospective field trial, we uncovered important differences in the association between vector-parasite distribution and human Chagas disease infection, which merit consideration in the design of screening programs for Chagas in Arequipa and other urban settings.

There are numerous reasons why the vector-based targeted screening strategy designed for a previously studied community (Guadalupe), yielded fewer cases when applied elsewhere in the city. In Guadalupe, parasite infections in both humans and vectors were clustered, and the age-prevalence curves suggested established epidemics of *T. cruzi* transmission [10], [31]. The present study sites, much closer to the city center, also contained clusters of parasite-infected vectors, but these consisted of fewer households. These smaller clusters are likely indicative of a relatively short history of vectorial transmission, leading to few locally acquired cases.

In addition to epidemiology and ecology, social and demographic phenomena may also affect patterns of human Chagas disease [35], [36]. Frequent migratory movement between rural and metropolitan Arequipa may bring parasite into the city [36], [37]; the cases of infection we detected were most associated with a history of triatomine exposure and residence in rural areas, where Chagas disease is historically endemic [4], [5], [6]. It is possible that the few clusters of parasite are due to introductions from outside that did not manage to spread beyond a handful of households in the new urban environment. That periurban time of residence did not significantly contribute to overall risk of infection in this study is another indication of minimal vectorial disease transmission in the study communities.

Interestingly, one of the 18 communities studied, Simón Bolívar, did display patterns of infection reminiscent of the micro-epidemic hotspots observed in Guadalupe. In Simón Bolivar, the 4 secondary cases detected within 15 meters of index cases may suggest significant rates of local vector-borne transmission at the time of insecticide application. The dissimilar migration history of the cases found in Simón Bolívar allow us to rule out that this clustering may be due to a group of infected migrants from the same sending community settling on the same city block, a phenomenon that has occurred in periurban settlements of Arequipa [37], [38]. Importantly, there was no obvious *a priori* evidence in Simón Bolívar to expect transmission to differ from the other 17 communities considered. This study adds to growing evidence of an uneven distribution of *T. cruzi* infection in the city of Arequipa [9], [10], not uncommon to this parasitic disease [39]. Other similar pockets of vector-borne transmission may exist in the city; the implementation of small pilot studies in infested areas followed by spatial adaptive sampling around human cases can help uncover them and determine the appropriate screening strategy for each setting. Where urban vectorial transmission is present, this adaptive strategy identifies secondary cases efficiently. When employed in an area without vectorial transmission, adaptive sampling should return few or no secondary cases, such that additional screening is curtailed and expenditures capped. Although harder to obtain, longitudinal entomological data, as opposed to the cross-sectional data utilized here, may be informative in locating these mini-epidemics. We are currently exploring community-based recognition and alert systems as a promising mechanism for obtaining this longitudinal vector data.

In addition, our disease screening activities took place between 5 months and 4 years after household insecticide application and collection of the entomologic data. It is not clear what the effect of this time lapse may be on the association between vector data and human infections detected. The strategies may work much better if these delays were eliminated. However, considering that exposure risk is greatly reduced by the elimination of vectors, we can reasonably expect that there were little new infections prior to our testing.

Another possible cause of error is the inability of migration histories to capture participants' short visits to endemic areas. While a potentially important source of exposure, there are methodological and recall challenges to documenting travel history at such a fine level. We found that screening based on personal risk assessment and residence-exposure history (ie, time lived in rural and/or infested areas) is advantageous for capturing high-risk individuals. Studies of blood donors in Canada and the US have found promising usefulness and operational feasibility of residence history questionnaires [26], [27], [40].

Our study consisted of operational research in a large heterogeneous city. In the wake of vector control campaigns, the target population was well-aware of their risk of Chagas disease. Residents of houses where no infestation was detected could have been at risk of vectorial transmission due to proximity to infested houses. However, during our fieldwork, it became clear that those who participated in this optional Chagas disease screening did so because they believed themselves to be at risk due to prior exposure. Participants from houses with no infestation detected often expressed concern about a previous infestation in their current or prior homes. In contrast, those who refused often reported being uninterested in testing because they did not consider themselves at risk, or because in their memory they had no contact with a vector. As a result, this study suffered from a self-selection bias according to perceived risk among participants living in vector-free houses at the time of the spray campaign. This bias likely caused an overestimate of the Chagas disease prevalence in houses with no infestation detected. Although not directly comparable, our observed overall prevalence of 2.28% is much higher than the 0.73% reported in a 2004–05 cross-sectional screening of pregnant women in Arequipa, which included populations from communities also studied here [41]. In contrast, we believe the prevalence estimate of 2.60% among the infected vector group to be quite accurate for persons living in this risk category due to the 98% participation rate.

Factors affecting participation need to be taken into consideration to design an economically optimal algorithm for targeted screening of Chagas disease. The high refusal rate among houses with no infestation detected required double the number of household visits per participant recruited than for the infected-vector group, making it the most expensive strategy in our cost calculation. If similar entomologic data from vector control campaigns are used to guide a human Chagas disease case-finding strategy, minimizing costs for fieldwork while still detecting cases would be optimal. Targeting screening according to the presence of *T. cruzi*-infected *T. infestans* was less expensive and similarly effective compared to the other strategies. Further, coupling screening to ongoing vector control campaigns can improve participation [19] with the added advantage of eliminating the time lapse between entomologic data and human testing that we experienced in some communities of this study.

In large cities like Arequipa, where *T. cruzi* exposure is highly heterogeneous, a targeted screening program is necessary for the prompt diagnosis of indeterminate Chagas disease, the prevention of future transmission and the maximization of limited resources [19]. A greater knowledge of the patterns of infection can be obtained by pilot studies, and would improve the design of screening strategies. Rural, urban and periurban places have different ecologies, and it is reasonable to expect different epidemiologies of Chagas disease in these settings [42]. The flexibility to adapt to the epidemiology that emerges during pilot screenings is key to an efficient case detection intervention. Finally, this data can help develop a brief residence history questionnaire for referring to diagnostic testing those at greatest risk of Chagas disease. Self-assessment of risk and triatomine exposure time is a potentially useful tool for screening; volunteer screening programs at local health posts or fairs may be very effective ways to capture infections in heterogeneous periurban communities subsequent to informative vector control campaigns.
